# Relevant Outcomes in Pediatric Acute Respiratory Distress Syndrome Studies

**DOI:** 10.3389/fped.2016.00051

**Published:** 2016-05-13

**Authors:** Nadir Yehya, Neal J. Thomas

**Affiliations:** ^1^Department of Anesthesiology and Critical Care Medicine, Children’s Hospital of Philadelphia, University of Pennsylvania, Philadelphia, PA, USA; ^2^Department of Pediatrics and Public Health Science, Division of Pediatric Critical Care Medicine, Penn State Hershey Children’s Hospital, Hershey, PA, USA

**Keywords:** pediatrics, ARDS, outcomes, acute respiratory distress syndrome, children, acute lung injury

## Abstract

Despite distinct epidemiology and outcomes, pediatric acute respiratory distress syndrome (PARDS) is often managed based on evidence extrapolated from treatment of adults. The impact of non-pulmonary processes on mortality as well as the lower mortality rate compared to adults with acute respiratory distress syndrome (ARDS) renders the utilization of short-term mortality as a primary outcome measure for interventional studies problematic. However, data regarding alternatives to mortality are profoundly understudied, and proposed alternatives, such as ventilator-free days, may be themselves subject to hidden biases. Given the neuropsychiatric and functional impairment in adult survivors of ARDS, characterization of these morbidities in children with PARDS is of paramount importance. The purpose of this review is to frame these challenges in the context of the existing pediatric literature, and using adult ARDS as a guide, suggest potential clinically relevant outcomes that deserve further investigation. The goal is to identify important areas of study in order to better define clinical practice and facilitate future interventional trials in PARDS.

## Introduction

Pediatric intensivists were not present for either the 1994 American-European Consensus Conference (AECC) (1) or the 2012 Berlin re-definition (2) of acute respiratory distress syndrome (ARDS), and so pediatric considerations were not addressed. Despite this limitation, AECC and Berlin definitions were historically applied to children without modification, despite the different epidemiology and outcomes of pediatric ARDS. To address this deficiency, the Pediatric Acute Lung Injury Consensus Conference (PALICC) was convened to propose specific definitions for pediatric acute respiratory distress syndrome (PARDS) (3). Notable differences in the PALICC definition are use of oxygenation index (OI) instead of PaO_2_/FiO_2_, the ability to diagnose PARDS in the absence of arterial blood gas analysis by using non-invasive measures of hypoxemia based on SpO_2_ [oxygen saturation index (OSI)], and less restrictive radiographic criteria.

Irrespective of definitions utilized, cohort studies and clinical trials have generally demonstrated lower mortality for PARDS (relative to adult ARDS), as well as an appreciable decrease in mortality over time (4, 5). Adult studies have demonstrated decreased pulmonary capacity, decreased quality of life, and worsened neurocognition among survivors of ARDS (6–8); however, comparable studies are lacking in PARDS. An appreciation of long-term sequelae is important for characterizing the epidemiology of this syndrome. Additionally, the already low and further decreasing mortality rate makes short-term survival an impractical endpoint for most clinical trials in PARDS, necessitating the identification of clinically relevant patient-centered outcomes to test future interventions. The purpose of this review is to identify the challenges in identifying appropriate outcomes for current and future studies in PARDS, framed in the context of the existing literature. Additionally, using adult ARDS studies as a guide, potential alternative outcomes that deserve further investigation in PARDS are suggested.

## Mortality

Pediatric acute respiratory distress syndrome has lower mortality than adult ARDS (4, 5), with mortality decreasing over time. Unfortunately, short-term mortality – such as 28- to 60-day mortality, pediatric intensive care unit (PICU) mortality, and hospital mortality – remains an objective, easily obtained, clinically relevant, and patient-centered outcome. As such, it is the most consistently reported endpoint in cohort studies (Table 1) (5, 9–23) and in clinical trials (Table 2) (24–32). However, predictors of mortality in PARDS are often not specific to PARDS but are characteristic of risk factors in several conditions that result in critical illness. Notably, immunocompromised status (5, 21, 26) and multisystem organ failure (MSOF) (5, 12, 17) are associated with increased mortality risk in several PARDS studies, including clinical trials (26). However, immunocompromised status and MSOF have little pulmonary specificity, are associated with mortality in sepsis, and are component variables of severity of illness scoring systems. Thus, a generalization of this observation states that children die *with* PARDS, rather than *because of* PARDS. In such cases, the associated PARDS has resolved at the time of death, despite the persistence of mechanical ventilation. Further complicating the use of mortality as an endpoint is elective withdrawal of potentially futile care, either for persistent MSOF, underlying refractory malignancy, or for presumed poor neurologic prognosis, none of which are specific for PARDS.

**Table 1:** **Reported outcomes in cohorts using PARDS-type inclusion criteria and ≥100 patients**.

Years	Centers	Countries	Cases	Outcomes	Comments	Reference
1986–1990	1	USA	100	Mortality (72%)	PaO_2_ ≤75 with FiO_2_ ≥0.5; bilateral infiltrates; 74% immunocompromised	(9)
1990–1991	14	France	123	Mortality (60%)	Intubated; FiO_2_ ≥0.5 on PEEP; bilateral infiltrates	(10)
1993–1995	1	Canada	131	Mortality (27%) Ventilator days survivors (8, range 1–27)	Intubated; PEEP ≥6 and FiO_2_ ≥0.5 for 12 h	(11)
1997–1998	10	USA, Canada	232	Mortality (40%)	Intubated; HFOV	(22)
1996–2000	2	USA	328	Mortality (22%) VFD (15 ± 11)	Non-invasive and intubated; AECC ALI with PF ≤300	(12)
2004–2005	12	Australia, New Zealand	117	Mortality (35%)	Non-invasive and intubated; AECC ALI with PF ≤300	(13)
2005–2006	26	China	358	Mortality (44%)	Any respiratory support; AECC ALI with PF ≤300	(14)
2000–2007	1	USA	398	Mortality (20%) VFD (17, IQR 0–24)	Intubated; PF ≤300; 192 with bilateral infiltrates	(15)
2007–2010	5	USA	168	Mortality (11%) VFD (20, IQR 10–23)	Intubated; AECC ALI with PF ≤300; excluded SCT	(16)
2009–2010	7	USA, Canada, Belgium, Switzerland, Netherlands	328	Mortality (38%)	Intubated; HFOV	(23)
2010–2011	21	Spain	146	Mortality (26%)	Intubated; AECC ARDS with PF ≤200	(17)
2009–2011	7	Italy, Spain, France, Austria, Netherlands	221	Mortality (17%) ECMO/death (19%)	Intubated; Berlin ARDS with PF ≤300; age 1–18 months	(18)
2009–2013	1	USA	312	Mortality (22%)	Intubated; Berlin ARDS with SpO_2_ cutoffs	(19)
2008–2014	5	USA	299	Mortality (13%)	Non-invasive and intubated; AECC ARDS with PF ≤200	(20)
2009–2014	12	USA	222	Mortality (60%) Ventilator days survivors (9, IQR 4–16)	Intubated; SCT	(21)
2011–2014	1	USA	283	Mortality (13%) VFD (17, IQR 3–21) Ventilator days survivors (10, IQR 7–17)	Intubated; AECC ALI and Berlin ARDS with PF ≤300	(5)

*AECC, American-European Consensus Conference; ALI, acute lung injury; ARDS, acute respiratory distress syndrome; HFOV, high-frequency oscillatory ventilation; PEEP, positive end-expiratory pressure; PF, PaO_2_/FiO_2_; SCT, stem cell transplant; VFD, ventilator-free days*.

**Table 2:** **Reported outcomes in select placebo-controlled randomized clinical trials in acute respiratory failure and PARDS**.

Trial	Years	Intervention	Total subjects	Primary outcome	Other reported outcomes	Reference
HFOV	1990–1994	HFOV versus conventional	58	Mortality (34% HFOV; 41% conventional)	Ventilator days in survivors; supplemental O_2_ requirement; chronic lung disease	(24)
Weaning protocol	1999–2001	Protocolized PSV or VSV versus no protocol	179	Days to extubation (median 2 for no protocol; 1.6 PSV; 1.8 VSV)	Successful extubation	(25)
Calfactant	2000–2003	Intratracheal calfactant versus air placebo	152	VFD (13 ± 8 for calfactant; 12 ± 11 for air)	Mortality; failure of conventional ventilation; oxygenation; supplemental O_2_ requirement; PICU and hospital LOS	(26)
Prone position	2001–2004	Prone versus supine position	101	VFD (16 ± 8 for supine; 16 ± 8 for prone)	Mortality; organ failure-free days; supplemental O_2_ requirement; PCPC; POPC	(27)
Inhaled nitric oxide	2003–2005	iNO versus nitrogen placebo	53	VFD (15 ± 8 for iNO; 9 ± 9 for placebo)	Mortality; ECMO/death	(28)
Lucinactant	2007–2010	Intratracheal lucinactant versus air placebo	165	Adjusted LSM of ventilator days (4 for lucinactant; 4.5 for air)	Mortality; VFD; PICU and hospital LOS	(29)
PED-CARDS	2008–2010	Intratracheal calfactant versus air placebo	109	Mortality (12% for calfactant; 9% for air)	VFD; oxygenation; PICU and hospital LOS	(30)
RESTORE	2009–2013	Protocolized sedation versus usual care	2449	Ventilator days (median 6.5 for protocol; 6.5 for usual care)	Mortality; duration of weaning; duration of non-invasive ventilation; PICU and hospital LOS	(31)
Steroids	2010–2014	Methylprednisolone versus placebo	35	Ventilator days (10 ± 7 for steroids; 10 ± 5 for placebo)	Mortality; VFD; oxygenation; PICU and hospital LOS	(32)

*ECMO, extracorporeal membrane oxygenation; HFOV, high-frequency oscillatory ventilation; LSM, least squares means; LOS, length of stay; PCPC, pediatric cerebral performance category; PED-CARDS, pediatric calfactant in acute respiratory distress syndrome; POPC, pediatric overall performance category; PICU, pediatric intensive care unit; PSV, pressure-support ventilation; RESTORE, randomized evaluation of sedation titration for respiratory failure; VFD, ventilator-free days; VSV, volume support ventilation*.

One example is worth examining in further detail. A multicenter randomized controlled trial (RCT) of exogenous surfactant (calfactant) in moderate and severe PARDS (OI > 7) demonstrated improved mortality associated with calfactant treatment (26). However, imbalance in the proportion of immunocompromised patients, with over-representation in the placebo arm, likely contributed to this effect, and after adjustment for immunocompromised status, the association between calfactant treatment and improved mortality was no longer evident (*p* = 0.07). Furthermore, patients received treatment within 48 h of intubation, but the proportion of patients successfully extubated did not differ between the groups and curves for cumulative successful extubation did not begin to diverge until 12 days after intubation, suggesting that factors unrelated to the initial PARDS insult, such as immunocompromised status, may have been responsible for mortality and prolonged ventilation. A follow-up trial of calfactant was restricted to immunocompromised children [Calfactant for Acute Lung Injury in Pediatric Stem Cell Transplant and Oncology Patients (CALIPSO)] using mortality as the primary outcome.

While mortality may be problematic as a primary endpoint for a general PARDS population, there remain subgroups of children with PARDS who still have a substantial mortality risk, yet with a reasonable chance of survival. CALIPSO is an example of limiting an intervention to a subgroup of PARDS with substantial mortality (>50%), albeit at the risk of difficult recruitment and reduced generalizability. Indeed, recently, successful trials in adult ARDS of neuromuscular blockade (33) and prone positioning (34) employed this strategy, as ARDS et Curarisation Systematique (ACURASYS) limited enrollment to patients with PaO_2_/FiO_2_ ≤150, rather than the typical ≤300. Prone position in severe ARDS (PROSEVA) required even more stringent enrollment criteria, as it requires PaO_2_/FiO_2_ ≤150 *after* 12–24 h of initial stabilization, thereby excluding patients who rapidly improved with standard ventilator management. In both cases, the goal was enrichment of a higher risk population in which the tested intervention could plausibly impact mortality with a reasonable sample size. This, simultaneously, avoids unnecessarily exposing patients to treatment when they have low risk of mortality and high probability of survival irrespective of randomization arm, thereby diluting any potential treatment effect. For PARDS to reproduce this, predictors of mortality risk need to be identified and validated. These predictors need to be available early in the PARDS course to allow enrollment within a timeframe amenable for interventions to work, ideally within 48 h of PARDS onset. This strategy has particular appeal for testing interventions for “refractory” PARDS, such as high-frequency oscillatory ventilation (HFOV), prone positioning, methylprednisolone, inhaled nitric oxide (iNO), and extracorporeal membrane oxygenation (ECMO).

## Duration of Mechanical Ventilation

Duration of ventilation is a common outcome described in PARDS studies, especially when this outcome is limited to survivors. This outcome has face validity, as more severe PARDS can reasonably be expected to require a longer duration of mechanical ventilation. The 2012 Berlin definition (2) demonstrated an increase in duration of mechanical ventilation in survivors across increasing severity classes of ARDS, which was confirmed in LUNG SAFE (Large Observational Study to Understand the Global Impact of Severe Acute Respiratory Failure) (35). This observation has been corroborated in PARDS when using oxygenation at 24 h, rather than at PARDS onset (5).

To be used as a valid endpoint, the definition of “duration of mechanical ventilation” needs to be limited to survivors, given the risk of contamination of this endpoint with non-survivors with a short-duration of ventilation. Alternatively, liberation from ventilation can be analyzed as the primary outcome of interest, with death treated as a competing outcome. Appropriate statistical techniques must be employed for quantifying the effect of an intervention while accounting for competing events (36). Furthermore, given the increased utilization of non-invasive ventilation both prior to (37–39) and after endotracheal intubation, duration of mechanical ventilation requires clear definition regarding whether non-invasive support is included. Both Berlin (mild) ARDS (2) and PALICC PARDS (3) definitions make allowances for non-invasive support, suggesting that screening for studies based on these criteria would allow for inclusion of a substantial number of non-intubated patients. This potential for increased enrollment needs consistent and well-delineated reporting of what is meant by “duration of mechanical ventilation.”

Therefore, while the endpoint “duration of ventilation in survivors” retains face validity and likely reflects the severity of PARDS, it is unclear exactly how “patient-centric” this outcome is. Specifically, it is unclear whether a given child would be better served with 10 days of invasive mechanical ventilation and extubated to high-flow cannula or whether 8 days of invasive ventilation followed by 4 days of non-invasive bi-level positive airway pressure (BiPAP) with full-facemask interface. Indeed, the answer likely varies between patients for a multitude of variables, including sedation requirements, strength, airway status, and indication for intubation.

Finally, duration of ventilation in survivors is complicated by the prevalence of subglottic stenosis, poor secretion tolerance, or severe upper airway obstruction from poor airway tone as an indication for prolonged intubation. Such patients may wean appropriately to minimal invasive support given their underlying PARDS severity, but the actual act of removing the endotracheal tube may be delayed, or ultimately attempted and unsuccessful, for reasons related primarily to their airway. Given the substantial number of comorbidities described in PARDS, reasons for prolonged intubation unrelated to the actual PARDS risk factor have the potential to confound the utility of duration of ventilation as an endpoint. An alternative has been proposed to only count the duration of time until successful completion of an extubation readiness test, irrespective of whether or not the patient is actually extubated (40). However, this has not been validated nor described in an actual practice or trial and does not address the prior criticism of not being patient-centered, as the child remains intubated.

## Ventilator-Free Days

Perhaps, the most commonly adopted surrogate endpoint in PARDS trials, recently (Table 2), is ventilator-free days (VFD) at some arbitrary endpoint (e.g., at 28 days). VFD at 28 days are typically derived by subtracting duration of ventilation in survivors from 28 and scoring non-survivors and those requiring ≥28 ventilator days as 0 (41). It has also been defined as “days alive and free of mechanical ventilation” (42), which creates confusion for cases where the patient is extubated on day 10, but dies on day 20 (10 days alive and free of mechanical ventilation is VFD = 10; non-survival at day 28 suggests VFD = 0). This composite endpoint combines mortality and duration of ventilation by penalizing non-survivors, unlike duration of ventilation. Similar to duration of ventilation in survivors, VFD at 28 days has demonstrated correlation across severity of Berlin ARDS (2) and PALICC PARDS (3) categories, with worse oxygenation categories associated with fewer VFD. This composite endpoint potentially represents efficiency, as an outcome of an intervention, which both reduces mortality and duration of ventilation, can be detected with a smaller sample size (42).

The same caveats regarding clarity of non-invasive support are required for VFD as mentioned for duration of ventilation (41). However, VFD has a major limitation as a composite endpoint, as the merged individual endpoints (mortality and ventilator duration) are *not* equivalent and interchangeable. A child requiring 30 days of mechanical ventilation, but surviving, cannot be considered identical to a child who dies after 7 days of ventilation, although both would be recorded as VFD = 0. Composite endpoints are best utilized when the separate endpoints are of equivalent importance for the patient, such as stroke or myocardial infarction in hypertensive adults. When initially described for adult ARDS, VFD was demonstrated to be useful only when the more pejorative outcome of mortality was improved alongside duration of ventilation (42). Given the >30% mortality in adult ARDS (35), this is a reasonable expectation: interventions which shorten ventilation *should* improve mortality, assuming mechanical ventilation and ARDS are in the causal pathway for non-survival. However, even in adults, this assumption can be problematic. The ARDSNet corticosteroid trial (43) failed to demonstrate superiority of methylprednisolone for persistent ARDS for the primary outcome of mortality at 60 days (29.2% mortality in methylprednisolone, 28.6% in placebo, *p* = 1). However, methylprednisolone treatment was associated with 4.4 additional VFD and 2.7 additional ICU-free days at 28 days. Significantly, more patients in the methylprednisolone arm required re-initiation of ventilation (28 versus 9%, *p* = 0.006). These discrepant results make interpretation of the trial difficult: mortality is reported at 60 days, but VFD at 28 days. Mortality is nominally higher in the methylprednisolone group, but VFD are also more favorable for methylprednisolone. Thus, in this case, the reporting of VFD offers no advantages or power relative to reporting on mortality alone: when an intervention has opposite effects on duration of ventilation and mortality, VFD merely confuses the interpretation.

In pediatrics, the utilization of VFD at 28 days is potential suspect for these same reasons, as PARDS mortality is much lower, and persistent hypoxemia is unlikely to be the cause of mortality. Thus, the effect on mortality is less certain to be in the same direction as duration of ventilation. For instance, a trial of ECMO for severe refractory PARDS may result in improved nominal mortality rates but would likely result in prolonged duration of ventilation, thereby complicating the interpretation and utility of VFD. Finally, several interventions sorely in need of testing in PARDS, including fluid management, sedation protocols, weaning, and extubation readiness all clearly impact length of ventilation much more so than they will impact mortality, hampering the utility of VFD as an outcome unless these parameters are protocolized in the context of the trial.

## Need for Extracorporeal Support as an Outcome

A more recently reported composite outcome for PARDS investigations has been the composite of need for ECMO or death (18, 28). This attempts to address the limitations of VFD and the low mortality (and thus difficult to adequately power) of PARDS. The underlying assumption is that lung injury severe enough to require ECMO is essentially refractory to conventional mechanical ventilation, and thus need for ECMO would be a death in any center unable to provide ECMO. Therefore, “ECMO” is close enough to “death” to justify combination as a composite endpoint.

The European Society for Pediatric and Neonatal Intensive Care (ESPNIC) used this definition to test the utility of the Berlin criteria in children (18) and demonstrated that the inclusion of a “severe” ARDS category improved validity with an increased risk of ECMO/death in children with Berlin-defined severe ARDS, whereas risks were similar when defined using AECC definitions. It should be noted, however, that the incidence of ECMO/death (18.6%) was only marginally increased over the incidence of mortality (17.2%), and that comparable analyses for the outcome mortality yielded identical conclusions.

A recently published RCT (28) for iNO (total *n* = 53) reported both mortality (28% placebo, 8% iNO, χ^2^ *p* = 0.07) and ECMO/death (48% placebo, 8% iNO, *p* < 0.01). The trial was powered for a difference in VFD at 28 days, for which it required a sample size of 169 children, and was stopped early for slow enrollment. Of note, the difference in the reported VFD in this trial was also significant. While the primary outcome of more VFD was achieved despite the small sample size, the reporting of ECMO/death in this study points to a potential mechanism, whereby iNO improved VFD. Specifically, exposure to iNO appeared to decrease the rate of ECMO utilization, suggesting an improvement in hypoxemia, thereby reducing total ventilator days and potentially impacting mortality. This is significant, as it implies a connection between improvement in hypoxemia and better outcomes in PARDS, a connection which is not consistently corroborated in adult ARDS trials (44). For certain trials of salvage therapy, such as methylprednisolone, iNO, prone positioning, and HFOV, the use of ECMO/death as a primary outcome may be rational. However, as in the example above, there is little information added by this specific reporting that was not also captured by the more conventional short-term outcome of VFD at 28 days. Additionally, as ECMO is not an outcome *per se* but simply an additional mode of supportive care, with subjective thresholds for its utilization among different centers and practitioners, the composite outcome of ECMO/death is difficult to standardize. Finally, the component variables of ECMO/death are not of equal importance to the patient, thus calling into question its validity as a patient-centered, clinically meaningful composite outcome.

## Post-Discharge Outcomes

There have been no studies examining long-term mortality in PARDS, but outcomes, such as 90-day, 6-month, or 12-month mortality, are unlikely to represent significant differences compared to short-term mortality. Additionally, long-term mortality is more likely to result from either the underlying condition or an unrelated cause and is unlikely to be a sequelae of PARDS. Therefore, alternative post-discharge outcomes are needed (Table 3). Recent attention has focused on the development of new morbidities in the PICU as a relevant outcome (45, 46), with up to twice the prevalence of mortality.

**Table 3:** **Potential outcomes for PARDS studies**.

Outcome	Timeframe	Advantages	Disadvantages
Mortality	Short term	Easy to obtain	Impractical given low baseline mortality
	28 or 60 days	Fixed time-point	
	PICU	Related to acute process	
	Hospital	Patient-centered	
	Medium and long term	Potentially captures longer period of risk for unfavorable outcomes	Similarly low rate
	90 days		Harder to obtain follow-up
	1 year		More related to underlying comorbidities
VFD	28 days	Easy to obtain	Imbalance in components of the composite outcome
		Increases power to detect clinically meaningful improvements related to shortened ventilation	Only increases power if intervention benefits both mortality and ventilator days
Ventilator days	28 days	Easy to obtain	Needs non-invasive support explicitly defined
	PICU LOS	Related to pulmonary nature of PARDS	Unclear if patient-centered
ECMO/death	Short term	Increases power to detect efficacy of pre-ECMO “salvage therapies”	Subjective use of ECMO
			Imbalance in components of the composite outcome
			Unclear if patient-centered
Neurocognitive and functional (POPC/PCPC)	Medium and long term	Rapid (POCP/PCPC)	More thorough cognitive function requires longer testing
	90 days	Patient-centered	
	1 year	Potentially completed over telephone	Changes with developmental age and with comorbidities
	Pre-return to school	Potentially more practical, as it is a prevalent outcome	
Pulmonary outcomes	Medium and long term	Patient-centered Related to pulmonary nature of PARDS	Requires infrastructure (expertise and equipment) for in-person follow-up
	90 days		
	1 year		
	Pre-return to school		
Biometric outcomes	Medium and long term	Patient-centered Does not require return to clinic Potentially improved response rate	Requires development, testing and validation Requires expertise HIPAA concerns Ownership concerns (who owns the data and how will it be used?)
	90 days		
	1 year		
	Pre-return to school		
Psychiatric	Long term	Patient-centered	Requires infrastructure (expertise) for in-person follow-up
		Potentially completed over telephone	
Health care utilization	Medium and long term	Patient-centered Does not require inpatient follow-up Related to pulmonary nature of PARDS Addresses cost to patient/family	Difficult to obtain Sensitive to local practices Potentially more related to underlying comorbidities than to PARDS
	90 days and 1 year re-hospitalization		

*ECMO, extracorporeal membrane oxygenation; LOS, length of stay; PARDS, pediatric acute respiratory distress syndrome; PCPC, pediatric cerebral performance category; POPC, pediatric overall performance category; PICU, pediatric intensive care unit; VFD, ventilator-free days*.

Few studies have investigated the physical or neurocognitive quality of life in survivors of PARDS (47–51). The existing studies are of extremely limited sample size (all *n* ≤ 11) and outdated, with ventilator management not reflective of current PICU practices (52, 53). In 1985, Fanconi et al. (47) published on pulmonary function testing (PFT) of nine survivors of PARDS ventilated between 1978 and 1982 (five of whom experienced peak pressures >40 cmH_2_O) at a mean 2.3-year follow-up. Seven of the nine were considered “hypoxemic,” with PaO_2_ <80 mmHg on room air, and eight of nine had ventilation inequalities on multibreath nitrogen washout. Increased peak pressures and increased exposure to FiO_2_ >0.5 during PARDS correlated with increased ventilation inequalities, suggesting a potential association between ventilator management and long-term pulmonary outcome. In a separate study published in 1996, 11 PARDS survivors ventilated between 1986 and 1993 (mean PaO_2_/FiO_2_ 160; 9 of 11 with peak pressures >40 cmH_2_O) with PFT performed at a mean 23-month follow-up demonstrated obstructive physiology in three children and mixed obstruction and restrictive physiology in an additional four children (49). The most recent investigation of PARDS survivors (51) occurred in children ventilated between 1986 and 1998 (all experienced pressure-controlled ventilation, with all peak pressures <35 cmH_2_O). These investigators were able to assess PFT in seven patients, finding one with an abnormal diffusion capacity, and a second with exercise-induced hypoxemia.

Based on these small case series, the PALICC group recommended that survivors of PARDS undergo screening for PFT abnormalities within 1 year of discharge (54). The small sample size of these existing studies, antiquated ventilator management, and variable follow-up time precludes any real assessment of the prevalence of pulmonary dysfunction in PARDS survivors. Larger scale, multicenter follow-up is sorely needed, potentially exploiting the infrastructure of existing pediatric critical care research networks and in collaboration with pediatric pulmonologists and rehabilitation providers.

Studies within this framework are becoming more common in pediatric critical care. The out-of-hospital arm of Therapeutic Hypothermia after Pediatric Cardia Arrest (THAPCA) trial (55) was powered for a primary outcome of a dichotomized (good versus bad) version of the Vineland Adaptive Behavior Scale, second edition (VABS-II). Ebrahim et al. (56) reported on the 1-month post-PICU admission outcome of 65 urgently admitted survivors using VABS-II, pediatric cerebral performance category (PCPC), pediatric overall performance category (POPC), and overall pediatric quality of life inventory, fourth edition. They demonstrated an overall poor quality of life for these patients at 1-month post-PICU admission. A recent review article identified potentially useful health-related quality of life (HRQL) metrics for pediatric critical care (57). This review identified substantial morbidity for PICU survivors, some of which were associated with treatments received during their PICU stay, suggesting possible modifiable risk factors. Additionally, a recent review has also suggested significant psychiatric morbidity in PICU survivors (58), including post-traumatic stress disorder (PTSD), depression, and behavioral disorders, with prevalence of post-traumatic stress symptoms potentially as high as 62% (59). Finally, the ongoing multicenter Life after Pediatric Sepsis Evaluation (LAPSE) study is a prospective observational study collecting information on quality of life, family dynamics and stress, and health care utilization in survivors of pediatric severe sepsis.

## Adult ARDS Investigations of Alternative Outcomes

Seminal work in adult ARDS long-term outcome (6, 7) has paved the way for potentially comparable studies in PARDS. In 2002, adult survivors of moderate and severe ARDS were followed at 3, 6, and 12 months, with a primary outcome of 6-min walk distance (6). The authors found that survivors of ARDS (median age 45 years) had persistent physical limitations at all time-points tested, primarily due to muscle wasting and weakness. At 12 months, only 49% of patients had returned to work. In multivariable regression, use of corticosteroids and duration of mechanical ventilation both negatively affected 6-min walk distance, suggesting a possible relationship between modifiable risk factors and medium-term functional outcome. In the subsequent 5-year follow-up study, the median 6-min walk distance remained below predicted values (7). However, pulmonary function had returned to near normal, and persistent exercise limitations were attributed to continuing weakness and neuropsychological impairments. Health-care costs continued to be substantial for survivors up to 5 years after discharge, especially in those with pre-existing comorbidities.

For PARDS investigators, this experience is instructive. The major strengths of these studies are the well-characterized, multicenter cohort, the longitudinal study design, the high rates of follow-up, and the in-person data collection. The granularity of the data allowed significant associations to be made regarding ICU exposures (e.g., corticosteroids) and subsequent medium- and long-term outcomes. While these observations remain hypothesis-generating, these are still essential initial steps toward determining how to design future prospective trials with clinically meaningful, patient-centered outcomes.

An earlier study employing an alternative design is also worth considering (60). A prospective case control interview/questionnaire study was performed of adult ARDS survivors matched with non-ARDS survivors with similar severity of illness at a median of 23 months after discharge. ARDS survivors demonstrated worse HRQL in nearly all domains tested, including respiratory-specific domains. The most profound reductions in ARDS survivors were in the domains assessing either physical limitations or on the impact of pulmonary symptoms on activities of daily living. This was the first study to assess the HRQL in ARDS survivors matched to similarly ill non-ARDS patients, thus minimizing the possibility that observations were simply reflections of severity of illness; rather, this study design increased the plausibly that these associations were either actually caused by having ARDS specifically, or conversely, by the treatments used for it.

The significance of long-term, patient-centered outcomes is elegantly made when considering the neuropsychological function in adult ARDS survivors of the fluid and catheter treatment trial (FACTT). The initial trial used a 2 × 2 factorial design to test (separately) the utility of pulmonary-artery catheters versus central-venous catheters, and the effects of a conservative versus a liberal fluid management strategy on hemodynamically stable ARDS patients (61, 62). The trial failed to demonstrate superiority of either fluid strategy in its primary outcome of 60-day mortality (25.5% mortality in fluid conservative, 28.4% in fluid liberal, *p* = 0.30). However, the conservative arm resulted in 2.5 more VFD (*p* < 0.001) and 2.2 additional ICU-free days (*p* < 0.001) (61) without additional increase in non-pulmonary organ failures. Based on these findings, the FACTT investigators recommended a conservative fluid strategy in hemodynamically stable ARDS patients.

The follow-up ARDS cognitive outcome study (ACOS) conducted telephone interviews of FACTT survivors at 2 and 12 months post-discharge (8). Similar to prior investigations (6, 60), the investigators found that most survivors (55–60%, depending on metric used) experienced long-term cognitive impairment. Interestingly, lower PaO_2_ (*p* = 0.015) and allocation to the conservative fluid arm (*p* = 0.005) were independently associated with long-term cognitive impairment. The PaO_2_ during ARDS reported in ACOS survivors with cognitive impairment was median 71 (interquartile range 67–80), well within the ARDSNet recommended PaO_2_ ranges of 55–80, suggesting that existing, arbitrary guidelines may be too permissive, and that this level of mild hypoxemia may be associated with long-term neurologic sequelae. Additionally, the conclusions of the FACTT trial that conservative fluid management resulted in 2.5 additional VFD without additional organ failures are now called into question, as 12-month neurologic function clearly suggests potential sub-clinical neurologic dysfunction, leading to long-term functional impairment. To date, no study in children with PARDS has investigated any sort of long-term outcome, and the efficacy of our interventions on long-term function in growing and developing children remains a mystery.

## Biometric Outcomes

One of the disadvantages of the existing framework for evaluating long-term outcomes is the expense and expertise necessary to bring back patients to a follow-up clinic and conduct PFT and neuromuscular testing. An alternative strategy has been demonstrated by cardiologists with the embrace of remote telemonitoring (RTM) technology. These have taken the form of devices, which record heart rate, cardiac rhythm, pulse oximetry, and blood pressure, with wireless transmission to a data collection center (63). Devices can be modified to also include brief questionnaires, adding additional data regarding subjective experiencing of symptoms by the patient. Home spirometry adapted with an automated modem for data transmission has been used in a trial for management of children with asthma (64). Device modifications exist, which can additionally measure grip strength, as well as assess flexibility and reaction time using game-playing scenarios (65), which may be able to address certain neurocognitive and neuromuscular outcomes in PARDS survivors. RTM devices have already been incorporated as interventions in clinical trials in adult heart failure (66, 67). The use of RTM for patients with pacemakers and implantable cardioverter defibrillators by pediatric cardiologists was associated with fewer clinic visits (68), providing proof of concept for RTM to allow follow-up for patients at lower cost.

Remote telemonitoring remains unexplored in pediatric critical illness research, although it possesses significant potential. Some of the limitations regarding patient loss to follow-up are nicely addressed by RTM. Platforms which may only require smartphones and appropriate adaptors, or which utilize gaming, are intuitively appealing to a pediatric population, and may improve compliance. However, expertise in development and interpretation are still necessary, and validation will be required prior to implementation.

## Conclusion

Mortality in PARDS is decreasing, and while it remains clinically relevant and patient-centered, it is impractical for most purposes, and its use should likely be limited to trials aimed at enrolling pre-determined higher risk groups. VFD is likely to remain the most common primary endpoint for clinical trials in the foreseeable future, but advocates should be aware of its limitations, and should ensure that the power of this composite outcome rests on whether the tested intervention improve *both* mortality and duration of ventilation in survivors. Finally, given the prevalence of long-term neuropsychiatric morbidity and functional impairment in adult ARDS survivors, it is imperative that these parameters are defined for children. After a better understanding of the burden of surviving PARDS on patients and families is obtained, studies can be designed to demonstrate a return to pre-morbid functioning, which is fundamentally most important to the child and family.

## Author Contributions

NY and NT researched the topic, reviewed the relevant source articles, and wrote the manuscript together.

## Conflict of Interest Statement

Dr. NT reports personal fees from Discovery Labs and Ikaria, and grants from the FDA, all outside of the submitted work. The remaining author declares that the research was conducted in the absence of any commercial or financial relationships that could be construed as a potential conflict of interest.
